# Dynamic scaffolds for neuronal signaling: in silico analysis of the TANC protein family

**DOI:** 10.1038/s41598-017-05748-5

**Published:** 2017-07-28

**Authors:** Alessandra Gasparini, Silvio C. E. Tosatto, Alessandra Murgia, Emanuela Leonardi

**Affiliations:** 1 Molecular Genetics of Neurodevelopmental disorders, Department of Woman and Child Health, University of Padua, Padua, Italy; 2 Department of Biomedical Sciences and CRIBI Biotechnology Center, University of Padua, Padua, Italy; 3 CNR Institute of Neuroscience, Padua, Italy; 4 Department of Neuroscience, University of Padua, Padua, Italy

## Abstract

The emergence of genes implicated across multiple comorbid neurologic disorders allows to identify shared underlying molecular pathways. Recently, investigation of patients with diverse neurologic disorders found TANC1 and TANC2 as possible candidate disease genes. While the TANC proteins have been reported as postsynaptic scaffolds influencing synaptic spines and excitatory synapse strength, their molecular functions remain unknown. Here, we conducted a comprehensive *in silico* analysis of the TANC protein family to characterize their molecular role and understand possible neurobiological consequences of their disruption. The known Ankyrin and tetratricopeptide repeat (TPR) domains have been modeled. The newly predicted N-terminal ATPase domain may function as a regulated molecular switch for downstream signaling. Several putative conserved protein binding motifs allowed to extend the TANC interaction network. Interestingly, we highlighted connections with different signaling pathways converging to modulate neuronal activity. Beyond a known role for TANC family members in the glutamate receptor pathway, they seem linked to planar cell polarity signaling, Hippo pathway, and cilium assembly. This suggests an important role in neuron projection, extension and differentiation.

## Introduction

Neurodevelopmental disorders (NDDs) are common conditions including clinically and genetically heterogeneous diseases, such as intellectual disability (ID), autism spectrum disorder (ASD), and epilepsy^1^. Advances in next generation sequencing have identified a large number of newly arising disease mutations which disrupt convergent molecular pathways involved in neuronal plasticity and synaptic strength^2–6^. In particular, scaffold proteins seem to play a critical role in glutamatergic neurotransmission, organizing different components of glutamate receptor complexes at post-synaptic densities (PSDs) and determining synaptic strength and plasticity^7^. Among these, the TANC1 and TANC2 genes, encoding for the recently described scaffold proteins, are emerging as candidate genes for NDD^8, 9^. TANC2 gene mutations were found in patients with different clinical conditions, ranging from ID and ASD to schizophrenia^3, 10, 11^. A case of *de novo* inversion encompassing TANC1, causing psychomotor-retardation was also recently reported in the literature^12^.

TANC1 interacts with PSD95, one of the most important and well characterized scaffold proteins, as well as additional postsynaptic proteins including glutamate receptors^8, 9^. The TANC proteins are expressed in the hippocampus and over-expression of either has been shown to increase dendritic spines and excitatory synapse strength in mice, although *in vivo* assays suggest differences in expression timing and knock-out phenotype. TANC1 reaches the highest levels in the adult brain and its depletion seems to impair spatial memory in mice. TANC2 is higher expressed during the early embryonic stages and seems to be involved in proper fetal development, with knock-outs causing *in utero* lethality^8^. Although available experimental evidence suggests an important role for these proteins in neuronal development, little is still known about the pathogenic mechanisms involved^8^.

The TANC1 and TANC2 proteins were named on the basis of their domain architecture, predicted to contain tetratricopeptide (TPR) and ankyrin (ANK) repeats as well as a coiled-coil domain^9^. Furthermore, a P-loop ATPase domain was first observed at the N-terminus of the rolling pebbles orthologs of TANC proteins by Leipe and colleagues using sequence profile analysis and sequence-based structure prediction to define the novel class of STAND (Signal Transducing ATPase with Numerous Domains) NTPase^13^. STAND proteins, unlike other NTPases, present a C-terminal helix bundle fused to the NTPase domain thought to transmit conformational changes due to NTP hydrolysis to downstream effector domains^13^. As an example, the closely related APAF1 protein is activated by the release of cytochrome *c*, which together with nucleotide binding, induces a conformational change in the P-loop ATPase driving apoptosome assembly^14^. Even though the nucleotide binding activity of the TANC P-loop domain and its functional role have to be demonstrated, this particular multi-domain architecture suggests at least a mechanistic similarity in molecular functions for TANC protein, combining a regulatory molecular switch with scaffold properties to assembly highly dynamic protein complexes.

In this work, we employed a combined bioinformatics strategy, integrating sequence and phylogenetic analysis with *in silico* modeling of structural domains to better characterize the structure-function relationship of the two TANC proteins. Furthermore, we conducted an in depth computational analysis to identify compositionally biased regions and candidate short linear motifs (SLiMs) in intrinsically disordered regions (IDR) of the proteins, which may provide further interaction surfaces mediating dynamic protein complex assembly. Experimental evidence for protein-protein interactions (PPI) in the literature or from PPI databases and predicted functional elements have been used to infer novel putative interactors for the two TANC members. Predicted and collected data highlight TANC involvement in orchestrating different neuronal signaling pathways, which may be implicated in the pathogenesis of diverse NDDs.

This analysis suggests structural and functional elements that will help the interpretation of newly discovered TANC mutations. It would be worthwhile to follow up experimentally to support the hypothesis of a functional mechanism for TANC as a dynamically regulated scaffold.

## Methods

### Sequence feature analysis

TANC1 and TANC2 (UniProt accession codes: Q9C0D5 and Q9HCD6, respectively) were downloaded from UniProt^15^, aligned using the MAFFT multiple sequence alignment software^16^ and visualized with Jalview^17^. Secondary structure was predicted using PSIPRED^18^, whereas domains, repeats and other features were predicted with InterproScan^19^. COILS^20^, MARCOIL^21^ and CCHMM-PROF^22^ were used to assess previously predicted coiled-coil regions, and TPR modules were predicted with TPRpred^23^. A repeat consensus was manually curated with Jalview from the MAFFT alignment. Further periodicities were searched with TRUST^24^, RADAR^25^ and Repetita^26^.

Regions outside predicted domains, as well as N- and C-terminal protein sequences, were assessed for intrinsic disorder, presence of compositionally biased regions (i.e., repeating amino acids) and short linear motifs (SLiMs) using MobiDB^27^ and ELM^28^. Since SLiMs have a high chance of random occurrence and their prediction often has low specificity, we selected for consideration only those mapping to disordered regions conserved among orthologs. Accessibility and localization in alternatively spliced regions are further evidences supporting the validation of the predicted SLiM (Gibson *et al*. 2015).

### Known TANC interactors analysis

A list of experimentally determined TANC interactors was compiled and manually annotated from the literature and the publicly available databases BioGrid^29^, IntAct^30^, and STRING^31^ (see Table 1). Three significant interactions (false positive rate <0.1) identified in the Cilium were also included^32^. Each interactor was annotated with its protein domain architecture and biological processes in which it is involved, retrieved from the InterPro^19^, UniProt^15^ and KEGG^33^ databases. Furthermore, PubMed was searched for papers describing the involvement of TANC in neuronal development using selected keywords. Interaction details (i.e. residues, sequence motif and domain) were manually curated from the literature.

**Table 1:** List of TANC interactors.

TANC	Interacting protein			Experimental evidence	Ref.	TANC interaction region
	Name	Domain architecture	Pathway			
1	α-internexin	Intermediate filament head, DNA-binding	Cytoskeleton organization	Co-IP	9	_
1	CAMKIIα	Kinase	Glutamate Receptor signaling	Co-IP	9	_
1	CASK	Casein Kappa	Glutamate Receptor signaling	Pull-down assay	9	_
2	CBY1	Chibby_fam	Wnt/Wingless signaling, Cilium assembly	SF-TAP/MS	32	_
2	CDC5L	myb- HTH DNA binding type 1 and 2, Myb/Cef1 domain	Spliceosome assembly	HTS AC-MS	76	_
2	CENPQ	CENP-Q domain, Coiled coil	Nucleosome assembly at the centromere	HTS AC-MS	77	_
2	CEP120*	2 C2, Coiled coil	Centrosome organization, Cilium assembly	HTS AC-MS	77	_
1	CEP128	Coiled coil	Centrosome organization, Cilium assembly	HTS AC-MS	78	_
1	CNTRL	4 LRR, 4 Coiled coil	Centrosome organization, Cilium assembly	HTS AC-MS	78	_
1	FBXW11	F-box, 7 WD repeats	Ubiquitin-mediated degradation	Co-IP	79	_
2	FMRP	Agenet-like, KH, FXMRP1_C_core, FXMR_C2	Regulation of translation	CLIP	83	_
1	Fodrin	23 Spectrin repeats, SH3,3 EF-hand	Cytoskeleton organization	Pull-down assay	9	ANK and TPR
1	GKAP	3 Coiled coil	Glutamate Receptor signaling	Co-IP	9	_
1	GluR1	TM	Glutamate Receptor signaling	Co-IP	9	_
1	GRIP	7 PDZ	Glutamate Receptor signaling	Pull-down assay	9	_
1	Homer	WH1/EVH1, Coiled coil	Glutamate Receptor signaling	Pull-down assay	9	_
2	INPP5E	13 repeats of P-X-X-P, Phosphatase	Cilium trafficking	SF-TAP/MS	32	_
2	LATS2	UB associated Kinase, AGC-kinase C-terminal	Hippo pathway	HTS PL-MS	74	_
2	MAPRE1	CH, EB1_C	Microtubule cytoskeleton regulation, Cilium assembly	SF-TAP/MS	32	_
1	MOV10	P-loop ATPase domain	RNA-mediated gene silencing	HTS AC-RNA	80	_
1	MINK	Kinase, CNH	Rap2-mediated signaling	Immunoblotting	75	TPR
1	NINL	4 EF-hand, 4 Coiled coil, KEN- box, D-box	Centrosome organization, Cilium assembly	HTS AC-MS	78	_
1	NR2B	Transmembrane receptor	Glutamate Receptor signaling	Co-IP	75	_
2	NR2C2	ZF- C4, NHR ligand binding	Nuclear receptor signaling pathways	HTS AC-MS	77	_
1	NXF1	RRM, 4 LLR repeats, NTF2, TAP-C	mRNA export from nucleus	HTS AC-RNA	80	_
2	PAK7	CRIB, kinase	Planar Cell Polarity pathway	HTS AC-MS	81	_
1	PCM1	Coiled coil, GTPase, molybdopterin domain	Centrosome organization, Cilium assembly	HTS AC-MS	78	_
2	PPP1CA	Ser/Thr phosphatase	Glutamate Receptor signaling, Hippo, Wnt signaling	HTS AC-MS	81	_
2	PPP1CC	Ser/Thr phosphatase	GluR, Hippo signaling	HTS AC-MS	81	_
1 & 2	PRICKLE1	PET, 3 LIMs	Planar Cell Polarity pathway	LC-MS/MS	72	_
1 & 2	PRICKLE2	PET, 3 LIMs	Planar Cell Polarity pathway	LC-MS/MS	72	_
1 & 2	PSD-95	3 PDZ, SH3, GK	Glutamate Receptor signaling	Y2H, Pull-down assay	8	LIG_PDZ_Class_1
1 & 2	SAP97	L27, 3 PDZ, SH3, GK	Glutamate Receptor signaling	Y2H, Pull-down assay	8	LIG_PDZ_Class_1
1	SCRIB	16 LRR repeats, 4 PDZ	Planar Cell Polarity pathway	SPR	65	LIG_PDZ_Class_1
1	SHANK1	6 ANK, SH3, PDZ, SAM	Glutamate Receptor signaling	Pull-down assay	9	_
2	SPIRE2	KIND, 3 WH2, ZF	Vescicle transport	HTS AC-MS	77	_
1	TNIK	Kinase, CNH	Rap2-mediated and Wnt signaling	Immunoblotting	75	TPR
2	XPO1 (CRM1)	Importin_N-term, 10 ARM/HEAT repeat like	Nuclear export	Pull down	82	_
2	YWHAB	14-3-3	Glutamate Receptor signaling, Hippo signaling	HTS AC-MS	74	_
2	ZYX	3 LIM, Zn binding	Hippo pathway	HTS AC-MS	81	_

For each interactor, the interacting TANC protein, the detection method and the binding region (experimentally validated) are here listed. Y2H: Yeast two hybrid; Co-IP: Co-immunoprecipitation; SPR: Surface plasmon resonance; HTS: High-Throughput System; AC: Affinity Capture; PL: Proximity Label; MS: Mass spectrometry; CLIP: Cross-Linking ImmunoPrecipitation; SF-TAP/MS: systematic tandem affinity purifications coupled to mass spectrometry. SLiMs are named according to the ELM nomenclature.

### TANC interaction prediction

TANC interaction predictions were made either for the binding site of known interactions or to infer novel interactors. For each interactor we searched for the putative domain or linear motifs predicted to mediate TANC interaction. We assume that if the known interactor is a class of protein or presents the domain known to bind a predicted TANC linear motif this may be the putative interactor binding site.

Collected PPI data and predicted binding sites/domains were used to infer novel putative interactors for the two TANC members. Proteins belonging to the same family usually interact in a similar way with a specific protein domain^34^. We assumed that when a protein has been found to interact with only one of the two TANC paralogs it is possible to infer it could interact with both paralogs ias long as they share a common conserved SLiM predicted to mediate this interaction.

### Mutation analysis

Pathogenicity of NDD associated variants in TANC proteins was assessed using twelve different prediction tools: Align-GVGD^35^, I-Mutant2.0^36^, MUpro^37^, MutationAssessor^38^, MutationTaster^39^, PhD-SNP^40^, Polyphen2^41^, PROVEAN^42^, SIFT^43^, SNAP2^44^ and UMD-Predictor^45^.

### Phylogenetic analysis

TANC orthologs were downloaded from OMA Browser^46^ to reconstruct the phylogeny of the protein family. Eighty one vertebrate sequences, representative of each infrasubphyla, were retrieved. Taking into account teleost lineage-specific genome duplication^47^, only one copy of each TANC protein was considered. The analysis comprised also earlier species in which duplication of TANC gene did not occurred: 2 arthropoda sequences (*Strigamia maritima* and *Ixodes scapularis*), 32 insect sequences, *Trichinella spiralis* (Nematoda), *Ciona intestinalis* (Tunicata). Multiple alignments were computed with ClustalO^48^ and manually curated using Jalview. Phylogenetic analysis and visualization were performed with MEGA6^49^, using Maximum Likelihood based on the JTT model + G (Gamma distributed Sites) with 500 bootstrap replicates.

### Homology modeling

The predicted domains were modeled separately in order to build more reliable models. Sequences for TANC1 and TANC2 domains were submitted to the homology detection method HHpred^50^. Multiple sequence alignment-based template detection was performed with HHblits (local alignment) against pdb70, taking into account also target-template secondary structure similarity (for details see Supplementary Table S4). The resulting target-template alignments were manually curated using the repeat consensus map and the consensus secondary structure prediction^51^, in analogy to our previous work^52^. Two models for each domain were built by homology with Modeller^53^ and their model quality was estimated with QMEAN^54^. The electrostatic surface of each model was calculated with Bluues^55^ and Consurf^56^ was used to map conservation for each residue based on OMA orthologs alignment. The structures were finally visualized using Pymol (DeLano Scientific LLC).

## Results

**Figure 1:**
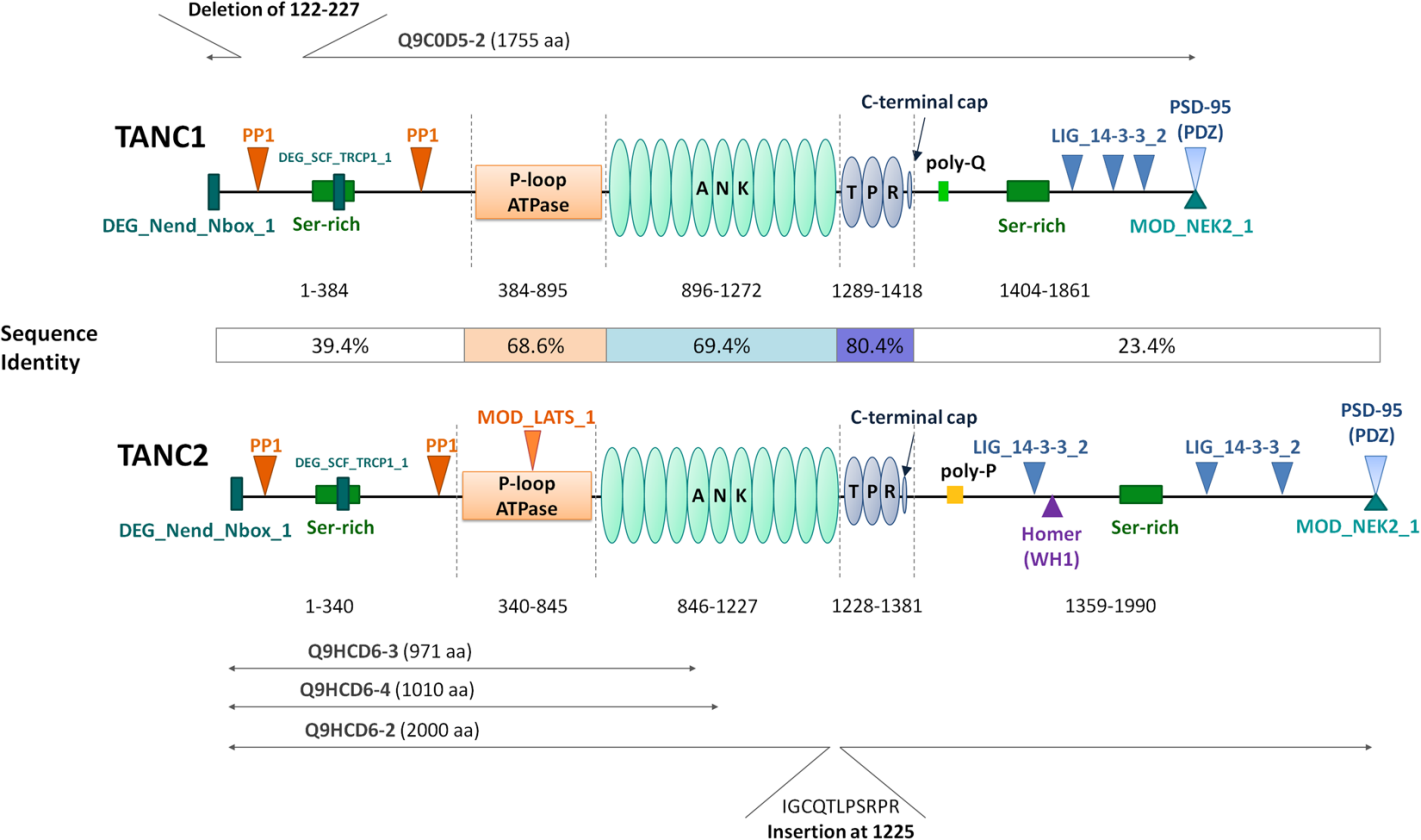
Sequence analysis of TANC proteins. An overview of TANC family domain architecture is here reported. Both TANC proteins are characterized by a putative P-loop NTPase domain (orange), an Ankyrin repeat containing domain (light teal) and a tetratricopeptide repeat region (blue). For each domain, the sequence boundaries and sequence identity between the two proteins are indicated. Conserved linear motifs are represented as follow: PDZ binding sequences (light blue triangles); PP1 docking motif (RVxF) (orange triangles); degrons (DEG_Nend_Nbox_1 and DEG_SCF_TRCP1_1) (deep teal rectangles); 14_3_3 binding sites (LIG_14-3-3_2) (blue triangles); Homer binding motif (LIG_EVH1_1) (Purple triangle); LATS1 kinase (light orange triangle); NEK2 phosphorylation motif (MOD_NEK2_1) (Teal triangle). Serine-rich regions are represented with green rectangles (TANC1 residues 170-243 and 1659–1689; TANC2 residues 125–189 and 1775–1865). The TANC1 glutamine-rich region (poly-Q) region and TANC2 glutamine/proline rich region (polyP) are in light green and yellow respectively. Alternative TANC protein isoforms are reported in grey. The TANC1 isoform Q9C0D5-2 (1755 residues) is missing the region 122-227. The TANC2 isoform Q9HCD6-2 is longer (2000 residues) due to an insertion at position 1225 (I > IGCQTLPSRPR). Q9HCD6-3 (971 residues) is truncated at residue 97 with different substitution in the region from position 944 to 971 (VDHLDKNGQCALVHAALRGHLEVVKFLI > VLAAQLCCFSSLFLYFRCILFLISSVTS). Q9HCD6-4 (1,010 residues) is truncated at residue 1011 with different substitution in the region from position 1006 to 1010 (IVSYL > VRSRQ).

Despite the emerging role of the TANC protein family in neuronal and embryonic development, little is known about their specific functions and molecular mechanisms^8^. A computational analysis of the TANC proteins starting from primary structure to explore the function of these twin proteins was thus performed. TANC1 and TANC2 are large proteins, of 1,861 and 1,990 residues respectively, sharing 51.9% overall amino acid identity, with similar multi-domain architecture (Table S1) resulting from an early duplication event (Supplementary Figure S1). InterproScan identifies two domains in both TANC protein sequences, an ankyrin (ANK) and tetratricopeptide repeat (TPR) domain (Fig. 1). An N-terminal P-loop containing nucleoside triphosphate hydrolase (NTPase) domain is predicted only in TANC2. The predicted domains are highly conserved among TANC paralogs. The N- and C-terminal disordered regions are quite variable. Crystal structures are not available for the TANC proteins, nor for any closely related proteins with similar domain architecture. To characterize the protein structure, each domain was modeled separately. The N- and C-terminal disordered regions were analyzed for the presence of a stretch rich in particular amino acid residues or conserved sequences containing predicted linear motifs likely to mediate protein interactions. Known interactors for both TANC proteins were downloaded from BioGrid^29^, IntAct^30^, and STRING^31^. Additional interactors were manually curated from the relevant literature (see Table 1). These findings were used to expand and curate a TANC protein interaction network (Table 2). While many interactors are in common between both TANC proteins, there are a two sets of proteins with experimental evidence for binding only one protein. In the following, we will describe each TANC region separately in more detail before using the predicted functional and structural elements to infer the possible impact of reported TANC2 missense mutations.

**Table 2:** Predictions for TANC interactors. For each interactor we searched for the putative domain or linear motifs predicted to mediate TANC interaction. We assume that if the known interactor is a class of protein or presents the domain known to bind a predicted TANC linear motif this may be the putative interactor binding site.

TANC	Predicted Interactor		Method	Predicted TANC interacting region
	Name	Domain architecture		
1	FBXW11	F-box, 7 WD repeats	TANC1 interactor, Conserved SLiM in IRD	DEG_SCF_TRCP1
				DEG_Nend_Nbox_1
1	YWHAB	14-3-3	TANC2 interactor, same SLiM in TANC2	LIG_14-3-3_2
1	PPP1CA	Ser/Thr phosphatase		DOC_PP1_RVXF_1
1	XPO1/CRM1	Importin_N-term, 10 ARM/HEAT repeat like		TRG_NES_CRM1_1
2	YWHAB	14-3-3	TANC2 interactor, Conserved SLiM in IRD	LIG_14-3-3_2
				LIG_14-3-3_3
2	PPP1CA	Ser/Thr phosphatase		DOC_PP1_RVXF_1
2	NR2C2	ZF- C4, NHR ligand binding		LIG_NRBOX (score 0,3)
2	XPO1/CRM1	Importin_N-term, 10 ARM/HEAT repeat like	TANC2 interactor, Conserved SLiM in P-loop domain	TRG_NES_CRM1_1
2	LATS2	UB associated Kinase, AGC-kinase C-terminal		MOD_LATS_1
2	Homer	WH1/EVH1, Coiled coil	TANC1 interactor	LIG_EVH1_1
2	SCRIB	16 LRR repeats, 4 PDZ	TANC1 interactor, same SLiM in TANC1	LIG_PDZ_Class1
2	FBXW11	F-box, 7 WD repeats		DEG_SCF_TRCP1
				DEG_Nend_Nbox_1
2	Fodrin	23 Spectrin repeats, SH3, 3 EF-hand	TANC1 interactor, same domain in TANC1	ANK and TPR
2	MINK	Kinase, CNH		TPR
2	TNIK	Kinase, CNH		TPR
1	CDK	Kinase	Conserved SLiM in IRD	MOD_CDK_1
1&2	G- Actin	Actin domain		LIG_Actin_WH2_2
1&2	Cyclins	Cyclin, N-terminal		DOC_CYCLIN_1
1&2	MAPK	Kinase		DOC_MAPK_gen_1
1&2	WW domain-containing protein	WW domain		DOC_WW_Pin1_4
1&2	Atg8 protein family	autophagy		LIG_LIR_Gen_1
1&2	CK1	kinase		MOD_CK1_1
1&2	GSK3	kinase		MOD_GSK3_1
1&2	NEK2	kinase		MOD_NEK2_1

SLiM: Short Linear motif; IDR: Intrinsically disordered region. SLiMs are named according to the ELM nomenclature.

### N-terminus

**Figure 2:**
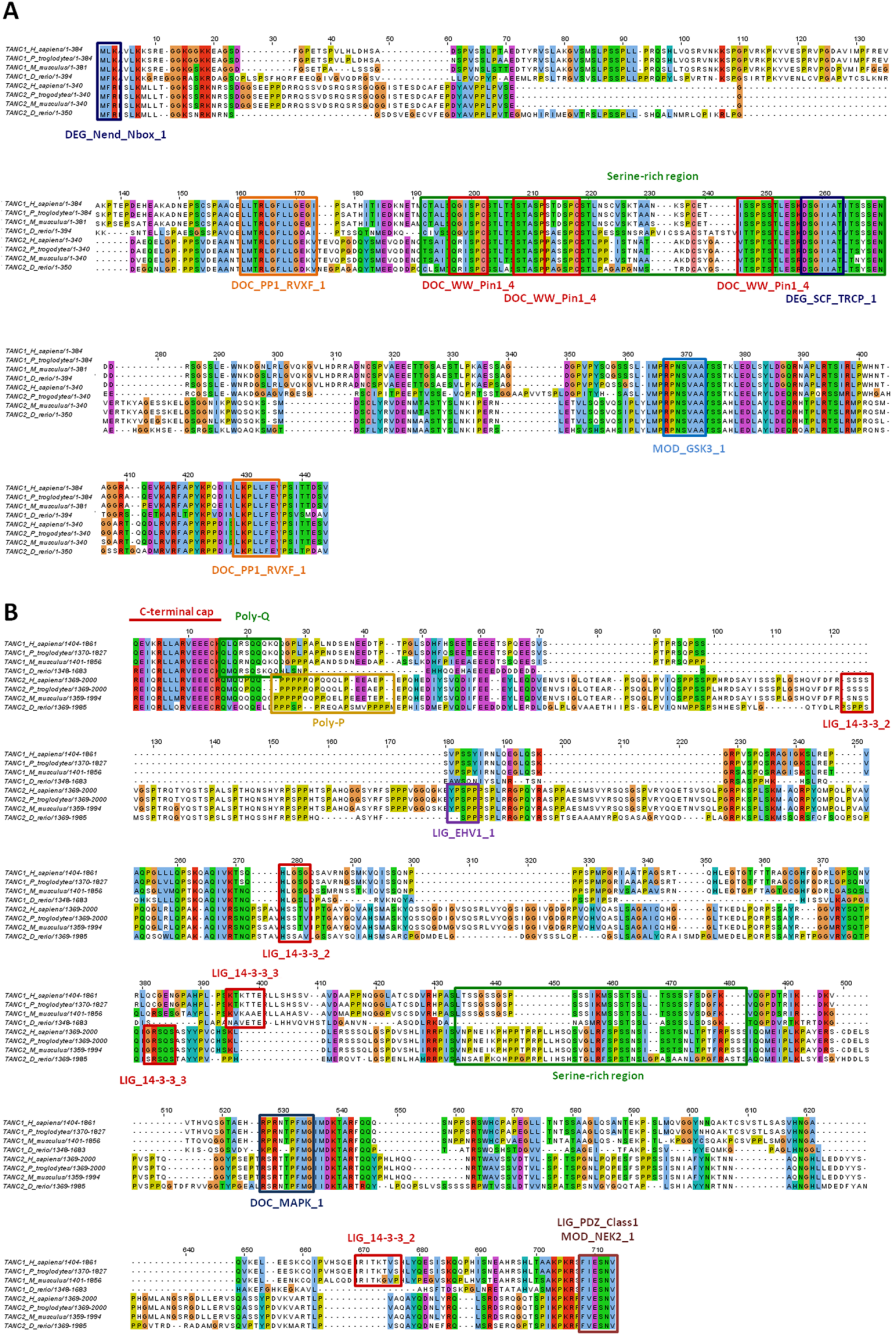
Multiple alignments of TANC N- and C- termini. Colour code based on Clustalx scheme. Linear motifs identified by ELM analysis are reported: DEG_Nend_Nbox_1: N-terminal motif that initiates protein degradation by binding to the N-box of N-recognins; DOC_PP1_RVXF_1: Protein phosphatase 1 catalytic subunit (PP1c) interacting motif; DOC_WW_Pin1_4: IV WW domain interaction motif; MOD_GSK3_1: GSK3 phosphorylation recognition site; MOD_NEK2_1: NEK2 phosphorylation motif; DEG_SCF_TRCP1_1: DSGxxS phospho-dependent degron recognized by F box protein of the SCF-betaTrCP1 complex; LIG_14-3-3_2: 14-3-3-binding motif; DOC_MAPK_1: MAPK docking motifs; LIG_PDZ_Class_1: PDZ-binding motif; LIG_14-3-3_3: 14-3-3-binding motif; LIG_EVH1_1: Proline-rich motif binding to signal transduction class I EVH1 domains. (**A**). N-terminus, (**B**). C-terminus.

The N- and C- termini are intrinsically disordered and share rather low identity between TANC paralogs, suggesting functional divergence (Fig. 1 and Fig. 2). A heterogeneous group of TANC2 sequences, comprising mammals, a bird and fish (*O. aries, M. putorius furo, M. domestica, F. albicollis, L. oculatus, O. niloticus*) defines the largest group sharing a 54 residue segment with TANC1 but no other TANC2 orthologs. A CK1 phosphorylation site and two SH3 binding motifs map to this sequence. This suggests that the N-terminal sequence was present in TANC1 first, duplicated in TANC2 orthologs and lost in other organisms, possibly to fine-tune the TANC2 interaction network (Supplementary Figure S1). An alignment of TANC sequences highlights the presence of short conserved sequences, shared across all members, containing putative linear motifs (Fig. 2). The N-terminus for instance presents two highly conserved motifs both involved in initiation of ubiquitin-dependent degradation and two protein phosphatase 1 (PP1) docking motifs (RVxF), almost identical in all considered sequences (Fig. 1 and Figure 2). Shared linear motifs also comprise several post-translational modification sites recognized by different kinases, such as GSK3, MAPK, and NEK2. These “hot spots” map in highly conserved serine-rich regions (SRR). The TANC1 isoform Q9C0D5-2 is missing residues 122–227, which contains the conserved PP1 docking motif and SRR, suggesting a regulatory role for these regions (Fig. 1).

### P-loop containing nucleoside triphosphate hydrolase (NTPase) domain

**Figure 3:**
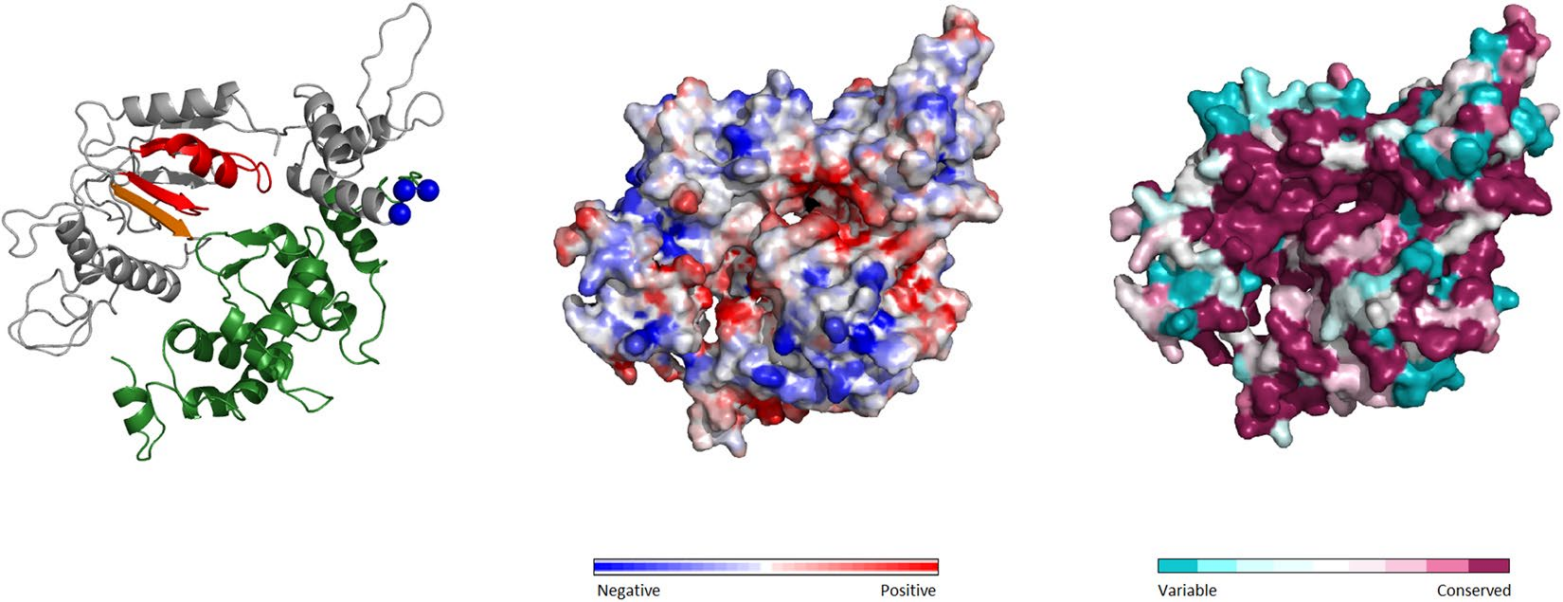
Structural analysis of ATPase domain in TANC1. Cartoon of TANC1 ATPase domain model (front part) is coloured as following: Walker motifs is in red, ASCE in orange, HETHS domain in green, GxP motif in blue spheres. Electrostatic properties of front surfaces are shown: negative charges in blue and red charges in red. ConSurf analysis of front surfaces, colour code from unconserved (cyan) to conserved (purple) residues.

The P-loop NTPase domain contains two sub-domains, the conserved NTPase α/β fold and a regulative region, known as helical third domain of STAND (HETHS). The NTPase domain of both TANC proteins was modeled considering both regions together. A HHpred search selected human apoptotic-protease activating factor 1 (APAF1; PDB code: 1z6t) as the best template, with 12.1% sequence identity for TANC1 and 12.5% for TANC2 (see Supplementary Table S1). Despite the presence of insertions between the TANC and template sequences, the conserved secondary structure elements superimpose well, especially in functional motifs on the catalytic core (Walker A, Walker B, and ASCE motifs). The Walker A and Walker B motifs define P-loop NTPase domains and are involved in nucleotide and Mg^2+^ cations binding respectively. The ASCE (“additional strand, catalytic E”) motif, typically situated between both Walker motifs, determines ATP as preferred substrate (Fig. 3). Moreover, residues placed in the catalytic pocket form a positively charged surface and are highly conserved in TANC orthologs (Fig. 3 and Supplementary Figure S2). While NTPase signature elements are rather conserved, the HETHS domain is quite variable among STAND family members and seems involved in family-specific regulative functions^13^. Indeed, since TANCs and APAF1 belong to different STAND NTPase families, their HETHS domains are more divergent in sequence and secondary structure. The 3D model quality evaluation of TANC2 and TANC1 is typical of more remotely homologous structures, with QMEAN scores of 0.421 and 0.391 respectively (Supplementary Table S1). However, lower quality regions are located in insertions corresponding to long disordered loops in TANC, while elements defining the catalytic core have low positional variability and higher reliability.

### Ankyrin (ANK) repeat domain

**Figure 4:**
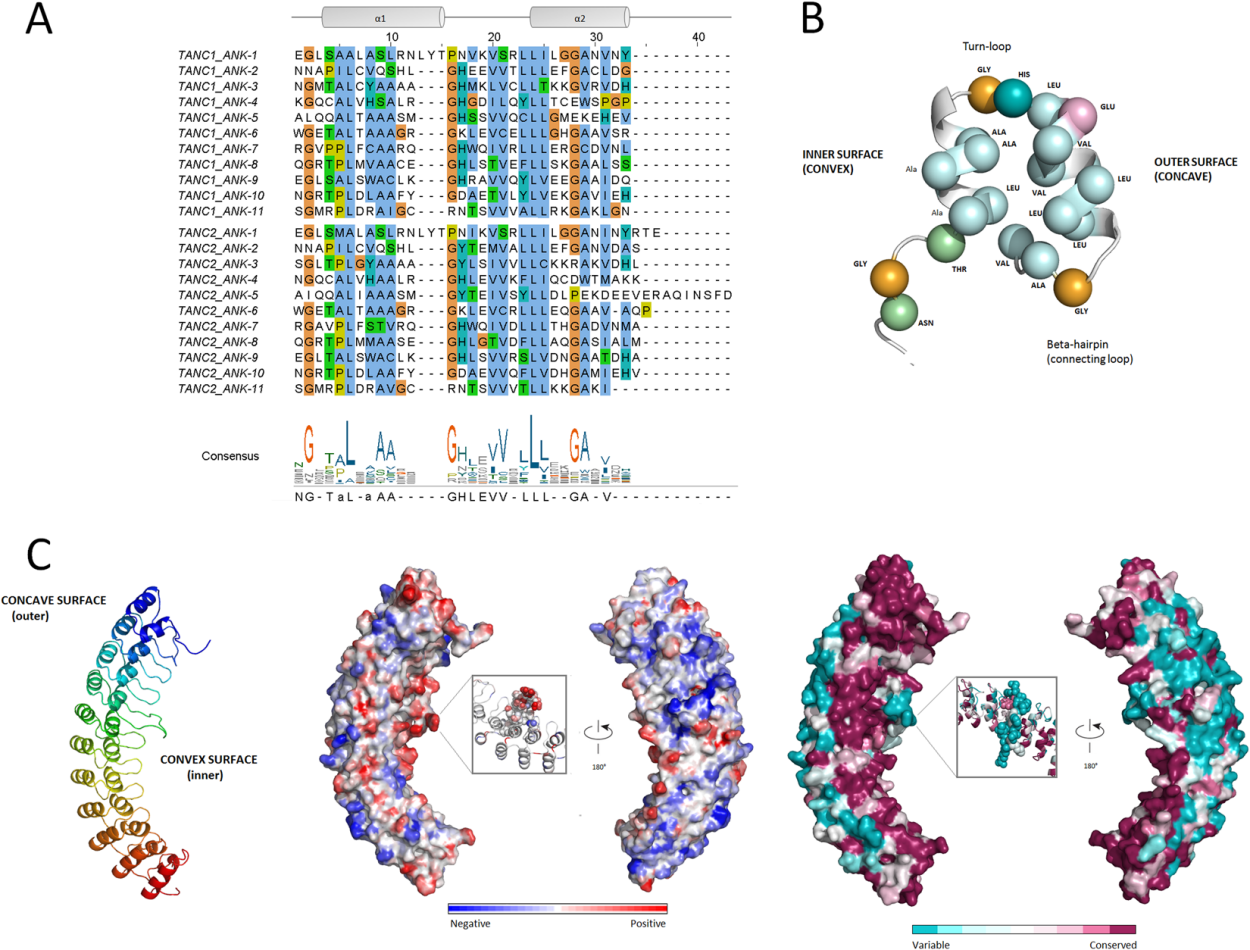
Ankyrin repeat overview and TANC1 Ankyrin domain model. (**A)** Consensus sequence of TANC ankyrin modules and related sequence logo. Residues that match the published consensus^57^ are reported in upper case. Secondary structure is shown above the alignment: the inner alpha helix (α1) and the outer alpha helix (α2) are connected by a turn-loop (black line). (**B)** Graphic representation of ANK repeats structure in TANC proteins. Conserved positions of ankyrin consensus pattern are reported in the diagram as spheres. Colour code refers to consensus logo: hydrophobic amino acids (A, L and V) are in light blue, glycine in orange, threonine and asparagine in green, histidine in teal, glutamate in violet, and proline in yellow. Residues matching the published consensus^57^ are reported in bold. (**C)** Cartoon of TANC1 AR domain model is coloured from N-terminus (blue) to C-terminus (red). Electrostatic properties of turn-loop surfaces and connecting-loop surfaces are shown: negative charges in blue and red charges in red. Consurf analysis of turn-loop surface and connecting-loop surface, colour code from unconserved (cyan) to conserved (purple) residues.

Ankyrin repeats are a relatively conserved motifs of ca. 33 residues with a consistent pattern of key residues essential for structural integrity (Fig. 4)^57^. The structural unit consists of a β-turn followed by two antiparallel α-helices and a loop connecting to the next repeat^57^. In both TANC proteins, eleven ankyrin (ANK) repeats are predicted by InterProScan. The alignment of ANK repeats reveals that the key conserved positions are overall maintained in both TANC1 and TANC2 (Fig. 4 and Supplementary Figure S3). Despite high sequence identity, the TANC1 repeat pattern is more regular, supporting divergent evolution. TANC2 presents longer loops and a peculiar negatively charged loop between the fifth and sixth repeat. Given its length, this loop separates the ANK domain into two regions and could be involved in TANC2 specific functions, as it is highly conserved among other species but not in TANC1 (Fig. 4).

For both TANC1 and TANC2, HHpred selected the human ankyrin-R (PDB code: 1n11_A) crystal structure with 12 ANK repeats as template. Using the same template should allow a more accurate identification of structural differences between both proteins. The HHpred alignments were manually refined using the previously defined ANK repeat alignment to maintain the structural integrity of each repeat. Both models have good quality, with QMEAN scores of 0.787 for TANC1 and 0.737 for TANC2. Each ANK domain is composed of eleven tandem repeats stacked together to form a linear solenoid structure. The linker loops of neighboring repeats are connected in a tail to head order to form a hairpin-like β-sheet usually involved in protein-protein interactions in most ANK proteins^58^. Conservation and electrostatic surface analysis highlighted specific features for each TANC protein (Fig. 4﻿ and Supplementary Figure S3). TANC2 presents higher overall conservation than TANC1, with a negative charge in the concave region compensated by the prevalently positive convex surface (Supplementary﻿ Figure S3). TANC1 presents a more significant separation between conserved residues belonging to the convex surface and unconserved positions in the concave region (Fig.﻿ 4C). The electrostatic surface follows the same pattern of TANC2, though more pronounced (Supplementary Figure 3). This region could be involved in electrostatic interactions with TANC binding partners.

### Tetratrico-peptide (TPR)-like repeat domain

**Figure 5:**
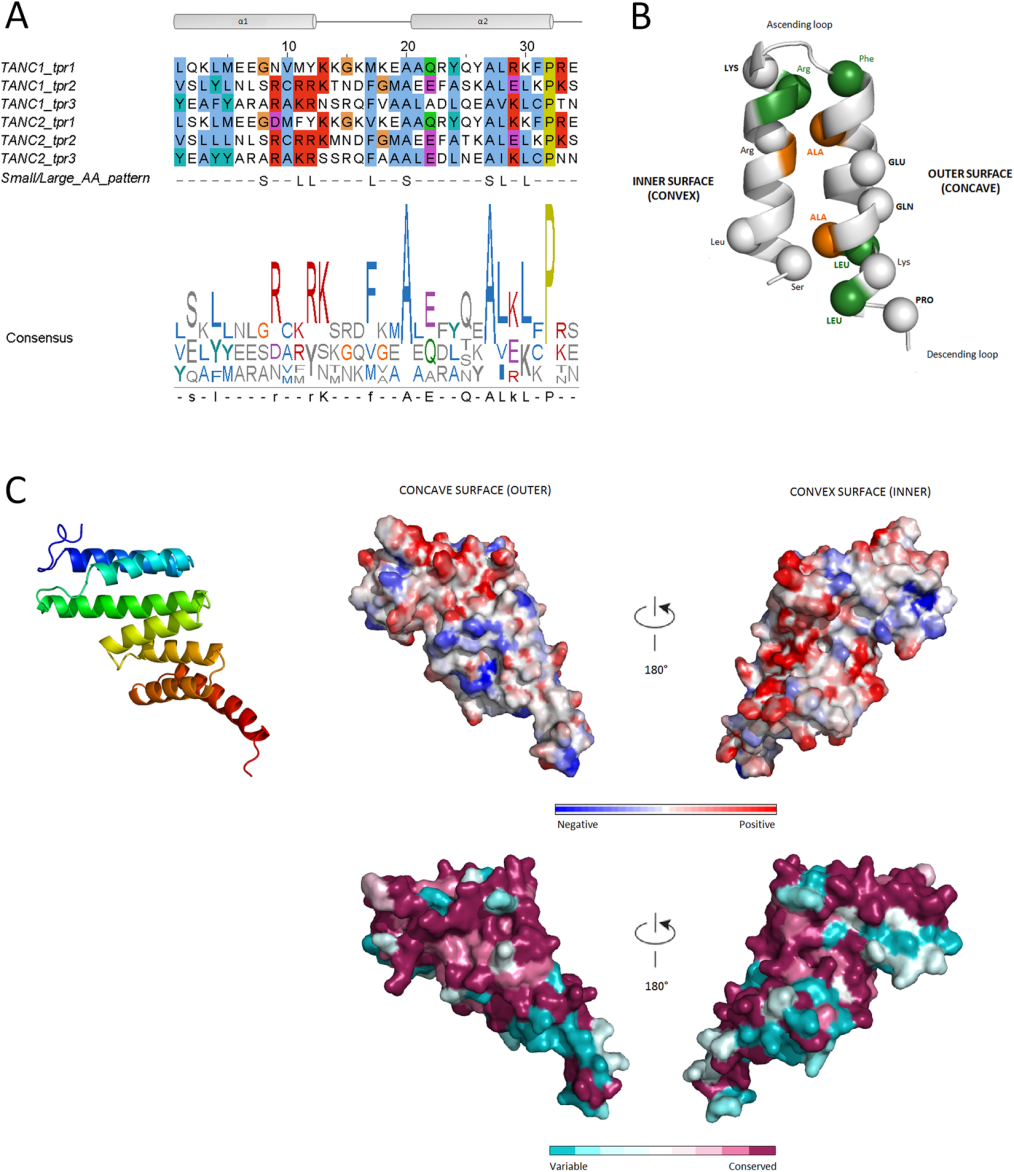
TPR repeat overview and TANC2 TPR model. (**A)** Consensus sequence repeat pattern of the TANC TPR domain and related sequence logo. Secondary structure is shown above the alignment: two alpha helices (grey shapes) connected by a loop (black line). Below the alignment, pattern of conserved small/large residues typical of TPR modules is reported: S indicates small residues, L for large residues. Residues that match the consensus are reported in upper case. (**B)** Graphic representation of repeats structure in TANC proteins. Conserved positions of TPR consensus pattern are reported in the diagram (spheres). Residues that match the consensus are reported in bold. Conserved small-large residue pattern is also represented: dark green for large residues and orange for small residues. (**C)** Cartoon of TANC2 TPR domain model is coloured from N-terminus (blue) to C-terminus (red). Electrostatic properties of concave and convex surfaces are shown: negative charges in blue and red charges in red. ConSurf analysis of turn-loop surfaces and connecting-loop surfaces, colour code from unconserved (cyan) to conserved (purple) residues.

Both TANC proteins are predicted to contain three TPR repeats which are extremely conserved among orthologous sequences. TPRs consist of 34 residues, whose consensus is defined by a pattern of small and large amino acids (Fig. 5). Each module is formed by two antiparallel α helices, forming a superhelical helix-turn-helix fold. TPRs are typically involved in protein-protein interactions and assembly of protein complexes^59, 60^. Despite the high sequence identity of human TANC TPR domains (80.4%), the template search selected different structures for homology modeling: human FK506-binding protein 52 (FKBP52, PDB code: 1P5Q) and *B. taurus* cyclophilin 40 (CYPD; PDB code: 1ihg) for TANC1 and TANC2 respectively (Supplementary Table S1).

The HHpred alignments were again manually refined using the previously defined TPR repeat alignment. QMEAN shows a rather high reliability for both models, with scores of 0.749 for TANC1 and 0.732 for TANC2. The TPR models were evaluated for both conservation and electrostatic properties (Fig. 5 and Supplementary Figure S4). ConSurf revealed the presence of highly conserved regions in the TPR domains corresponding to the convex surfaces, with prevailing positively charged surfaces in both TANC proteins. On the other hand, the concave part seems to be less conserved, with the exception of negatively charged residues at the C-terminus.

A coiled-coil region was previously thought to map downstream from the TPR domains^9^. Unlike TPRs, most domain predictors did not recognize any significant coiled coil region in TANC proteins (Supplementary Table S2). The presence of coiled-coil structures was assessed using three different tools. In both TANC sequences, all coiled coil predictors recognize a region downstream of the TPR-region with a low reliability score (Supplementary Table S2). However, secondary structure and a further manual evaluation of coiled coil motifs do not support this prediction^61^. Only one helix could be recognized downstream from the TPR domain for both TANC proteins (Supplementary Table S3). To exclude the presence of degenerate repeats in this position, TPR prediction was performed using TPR-pred. The analysis highlighted a low confidence TPR module (P-value > e-03) in TANC1, but not in TANC2 (Supplementary Table S3). We conclude that the helix is neither a coiled coil nor a TPR repeat, but may represent a C-terminal cap for the TPR domain. Similar C-terminal capping structures consisting of a 22 residue helix stabilizing the TPR fold^59, 62^ are present in both TANC1 and TANC2.

### C-terminus

In each TANC protein, the C-terminal region is preceded by the TPR C-capping helix and ends with the final PDZ binding motif. The Q9HCD6-3 and Q9HCD6-4 TANC isoforms are missing most of the ANK domain and the C-terminus, with the third - fourth ankyrin repeats and the fifth ankyrin repeat modified, respectively. Only few sequence stretches in the C-terminus have a significant similarity between TANC proteins and their orthologs (Fig. 1 and Fig. 2). As expected, ELM recognized the highly conserved PDZ-binding motif in both paralogs, which has been demonstrated to mediate TANC interaction with PSD95, SAP97 and SCRIB^8, 65^. A poly-glutamine region followed by a proline stretch and a serine-rich region (SRR) are present in both TANC C-termini. Furthermore, MAPK and WW binding sites are predicted in the C-terminus of all TANC homologs (Fig. 2). These sites are partially overlapping and located in a region predicted to be phosphorylated by different kinases. Several 14-3-3 binding motifs are also predicted on different positions in the TANC C-termini.

The TANC2 C-terminus, but not its paralog, presents an unusual number of 27 conserved tyrosine residues showing a periodicity of ca. 12 residues. The presence of possible repetitive modules was therefore assessed. As expected, no repeat pattern was identified for TANC1, whereas both TRUST and RADAR recognized four repetitive regions in the sequence preceding the SRR. Further manual curation of TANC2 repeats suggests the presence of shorter modules of approximately twelve residues, in which the tyrosine residue represents the main signature. Taken together, these findings confirm the presence of a regular pattern that could organize the C-terminus and have a regulative role in protein function.

### TANC network

We manually curated 24 TANC1 and 20 TANC2 interacting proteins. Thirteen TANC1 interactors and five TANC2 interactors were retrieved directly from publications. The remaining interactors have been determined by High-Throughput Screening (HTS) methods and deposited in publicly available PPI databases. The TANC interacting regions have been experimentally determined for only six TANC1 and two TANC2 interactors (see Table 1). Three PDZ domain proteins interacting with the C-terminal PDZ binding motif in TANC are considered mutually exclusive.

For one known TANC1 and five TANC2 interactors we predicted a putative interacting site. These proteins present a domain or belong to a class of proteins, which may recognize a conserved linear motif mapping in a disordered TANC regions. Exportin-1 and LATS2 have a predicted binding motif on the structured TANC2 ATPase domain. These motifs are located in loops that may be exposed upon conformational changes of the domain.

We inferred novel interactors for each TANC member based on known interactors and shared conserved linear motifs of the paralog (see Table 2). Three TANC1 interactors may also bind TANC2 through shared linear motifs. The three proteins found to interact with the globular domains of TANC1 (Fodrin, MINK, and TNIK) may also bind TANC2, although surface analysis of the ANK and TPR domains did not highlight a common conserved region.

Finally, the N- and C-termini of both TANC proteins contain shared conserved binding motifs for different kinases and WW domain proteins. We hypothesize that these proteins may mediate post-translational TANC modifications.

### Missense mutation analysis

Three TANC2 missense mutations have been reported in three unrelated patients with different neuropsychiatric phenotypes^3, 11, 63^. The two variants p.Arg760Cys and p.Ala794Val map on the ATPase regulative domain. The former has been found *de novo* in a pediatric patient presenting intellectual disability^63^. The p.Arg760Cys variant maps on a buried loop facing the ASCE strand within the ATPase regulative domain in catalytic pocket. The substitution of a charged arginine residue with a cysteine may have some effect on the catalytic pocket, where charged residues coordinate Mg^2+^ ions and binding of ATP molecules. The p.Ala794Val was inherited from the father in a patient with schizophrenia^3^. It affects a buried residue in the ninth helix of the regulative region that could affecting folding due to steric clashes. Both mutations are predicted as pathogenic by most prediction tools (11/12 for R760C and 12/12 for A794V, details in Supplementary Table S4) and likely affect regulative domain stability and ATPase activity. A third inherited mutation mapping to the C-terminal tail (p.His1689Arg) was found in a patient with autism spectrum disorder^11^. Although it maps within a conserved region, the Histidine to Arginine substitution is only predicted to be damaging by six of twelve tested methods (Supplementary Table S4).

## Discussion

Recently, evidence from mouse models and human patients suggested the TANC proteins as candidates for NDD. Despite different expression profiles in the brain, TANC1 and TANC2 have both been shown to positively regulate dendritic spines and excitatory synapses^8^. The TANC family has been described as PSD95 partners found to localize and interact with several postsynaptic proteins^9^. Here, we report an in depth *in silico* analysis of the TANC family structure and function to gain insights on their molecular function as well as to elucidate the role of these proteins in NDDs. The P-loop domain model suggests that the TANC proteins may have an ATPase activity since all functional elements are conserved, although the regulative domain differs from other proteins of this class and its role has to be demonstrated. Modeling the repeat domains allowed identifying conserved PPI interfaces for both ANK and TPR domains, with different electrostatic charges possibly involved in protein binding. Despite previously reported predictions, sequence and structural analysis of the TPR domain allowed to exclude the presence of coiled-coil region in TANC, as the mispredicted region corresponds to a stabilizing C-capping element of the TPR domain.

**Figure 6:**
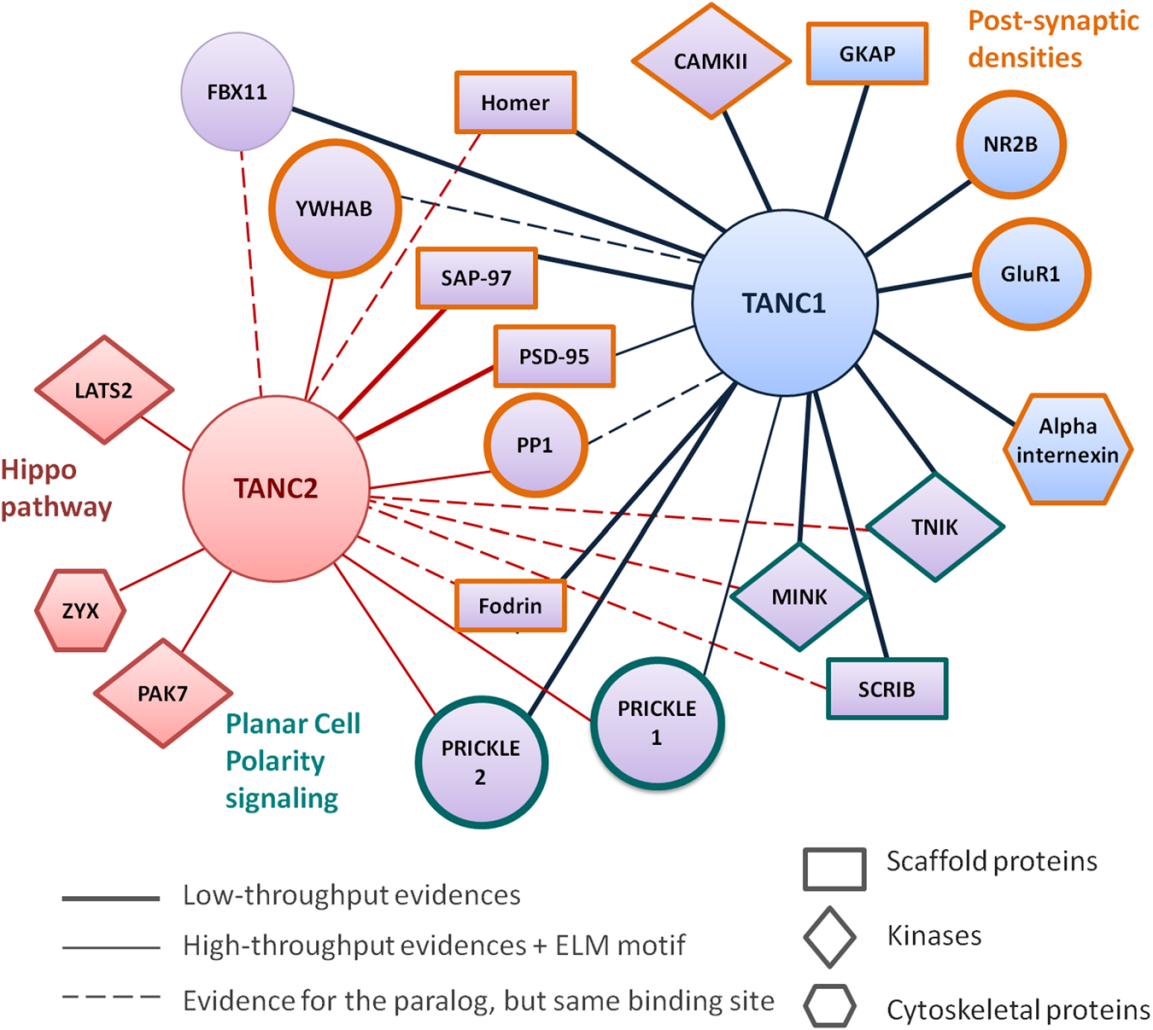
TANC protein interaction network. TANC interaction partners identified by low throughput data (solid lines), PPI database evidence (thinner lines) or linear motifs prediction. Interactions that are proved only in one paralog, but mediated by binding sites (linear motif or structural domain) that are identical in both proteins, are reported as dotted edges. TANC1 interactors only are colored in light blue; TANC2 interactors only in red: while TANC interactors both are in violet. Interactors are represented with different shapes based on specific molecular function: scaffold proteins (rectangles), protein kinases (rhombus); cytoskeleton proteins (hexagons). TANC proteins are connected with different neuronal regulative proteins, belonging to Planar Cell Polarity signalling (teal outline), Hippo pathway (dark red outline) and glutamate signalling (orange outline).

Along the N and C- terminal disordered regions of TANC we predicted several conserved SLiMs supporting interactions from high-throughput experiments known to have false positives (Table 2 and Fig. 6). The prediction of putative interacting regions, besides inferring novel interactors, allowed to define some proteins as mutually exclusive interactors. PSD95 and SCRIB interact with the TANC PDZ linear motif anchoring TANC proteins to the glutamate receptor or in PCP signaling^8, 9, 64–66^. Although most short motif patterns have a high chance of random occurrence and their prediction may have low specificity, we used stringent criteria^67^ to select putative protein binding sites. To be considered, a binding site has to be conserved among orthologs, or shared among paralogs, and mapping to a disordered region. Alternatively spliced regions are also favorable factor of being a true binding site. Moreover, the putative motif is supported if its binding protein is a known TANCs interactor or is involved in the same biological processe^67^.

We expanded the functional network of TANC proteins, integrating prediction and high throughput data, and inferring protein partners based on information of one of the two TANC family member (see Fig. 6). We found that the TANC N-termini present several conserved linear motifs, which may be involved in a broader range of cell regulation, including phosphodegrons and phosphatase docking motifs. These motifs could be the target of two TANC interactors identified by high-throughput screening, protein phosphatase 1 (PP1) and FBXW11. The latter is a component of the SCF E3 ubiquitin-protein ligase complex implicated in recognition of phosphorylated proteins targeted for degradation^68^. PP1 is one of the three phosphatases expressed in neurons regulating NMDAR-dependent Long Term Depression (LTD) during development^69^.

Another mechanism involved in functional TANC regulation is suggested by the findings that RNAs of both TANCs are targets of the MOV10 RNA helicase and the Fragile X mental retardation protein (FMRP)^70, 71^. Recently, MOV10 was found to be a functional partner of FMRP^71^. MOV10 promotes miRNA-mediated translational suppression of its target RNAs, while FMRP regulates synaptic strength at glutamatergic synapses by controlling translation of specific RNAs.

TANC regulation may also occur through post-translational modifications (PTM) sites we have predicted. Different kinases, such as CAMKII, MINK TNIK, PAK7, LATS2, have been identified as TANC interactors and PTM sites have been frequently shown to conditionally switch motif-mediated interactions^28^ triggering different signaling pathways. Predicted and collected PPI data allowed us to position TANC proteins in several biological processes, other than post-synaptic density proteins, such as the planar cell polarity pathway^72, 73^, Wnt signaling and Cilium assembly. We also found for TANC1 and TANC2 specific connections with Rap2-mediated and Hippo signaling^74^, respectively, that may explain different roles of TANC1 and TANC2 in brain function. However, all of these pathways contribute in different ways to correct neuronal development and maintenance^65, 66, 72, 75^.

The TANC proteins thus appear to be regulated at several levels from synthesis to degradation, while being involved in pathways controlling neural development and maintenance. It is likely that alterations of these proteins may affect different processes, thus explaining the broader range of disease phenotypes associated with TANC variants.

The performed analysis allowed us to discover structural and functional elements that will help the interpretation of newly discovered TANC gene variants. It would be worth following them up experimentally to support a mechanistic model for TANC function as a dynamically regulated scaffold.

## Conclusions

Here, we report a comprehensive *in silico* analysis of the TANC proteins to better characterize their molecular role in neurons. Domain architecture analysis of TANCs predicts a distinct ATPase domain that may confer the ability to function as regulated molecular switches. Future experiments will have to prove that TANCs have nucleotide binding activity. This mechanistic aspect can be easily used to turn off some signals and trigger others in different pathways. TANCs were found implicated in different neuronal pathways, including glutamate receptors, planar cell polarity and Cilium assembly. All of these converge to modulate neuron projection development and synaptic plasticity. However, it seems that only TANC2 is involved in Hippo signaling, which is linked to neurite growing and branching. This finding may explain the differential role of TANC2 in early embryonic development.

## Electronic supplementary material

Supplementary Material
