# Supplementary material for: Dynamic scaffolds for neuronal signaling: in silico analysis of the TANC protein family
Source: Sci Rep. 2017 Jul 28;7:6829. doi: 10.1038/s41598-017-05748-5 (PMC5533708; doi:10.1038/s41598-017-05748-5)
Supplement: Supplementary file 1 — Supplementary Material [file 41598_2017_5748_MOESM1_ESM.pdf]

Dynamic scaffolds for neuronal signaling: in silico analysis of the TANC protein family

Alessandra Gasparini[1,2], Silvio C.E. Tosatto[2,3,*], Alessandra Murgia[1], Emanuela Leonardi[1,*]

1. *Molecular Genetics of Neurodevelopmental disorders, Department of Woman and Child's health, University of Padua*
2. *Department of Biomedical Sciences and CRIBI Biotechnology Center, University of Padova*
3. *CNR Institute of Neuroscience, Padua*

** corresponding authors: silvio.tosatto@unipd.it , emanuela.leonardi@unipd.it*

Supplementary Material

Supplementary Tables

Target	Template	Prob	E-value	P-value	Score	SS	Cols	Query HMM	Template HMM	Id%	Qmean
TANC1_ATPase	1z6t	99.9	8.3e-23	2.3e-27	219.2	22.7	344	1-495	126-504 (591)	12.1%	0.391
TANC1_ANK	1n11	100.0	4.7e-65	1.3e-69	446.4	41.8	355	8-376	17-371 (437)	29.1%	0.787
TANC1_TPR	1p5q	99.9	1.1e-19	2.9e-24	122.0	18.6	139	1-155	134-287 (336)	23,9%	0.749
TANC2_ATPase	1z6t	99.8	2.3e-19	6.4e-24	192.0	23.2	344	1-483	126-498 (591)	12.5%	0.421
TANC2_ANK	1n11	100.0	5e-65	1.4e-69	447.8	39.0	355	8-381	17-371 (437)	28,10%	0.737
TANC2_TPR	1ihg	99.9	7.5e-20	2.1e-24	122.3	18.7	138	3-153	209-362 (370)	23.5%	0.732

Supplementary Table S1: HHpred template search results. Details of best hits for the best template of TANC domains are reported. **Prob**: Probability of template to be a true positive. **E-value**: expect-value. (average number of false positives) **P-value**: It is the probability that in a pairwise comparison a wrong hit will score at least this good. **Score**: raw score. calculated by comparing the amino acid distributions between columns from the query alignment and columns from the template alignment. The probabilities for insertions and deletions at each position in the alignments are taken into account as position specific gap penalties. **SS**: Secondary structure score. Cols: The number of aligned match-match columns in the HMM-HMM alignment. **Query HMM**: Range of query match states aligned **Template HMM**: range of template match states aligned and, in parenthesis, total number of match states in template HMM. **QMEAN**: global score of the whole model reflecting the predicted model reliability ranging from 0 to 1. **1z6t.pdb**: Structure of the apoptotic protease-activating factor 1 bound to ADP; **1n11.pdb** : D34 region of human ankyrin-R and linker; 1p5q.pdb: Crystal Structure of FKBP52 C-terminal Domain; **1ihg.pdb**: Bovine Cyclophilin 40, monoclinic form.

UniProt ID	TPRs position	MARCOIL Position		MARCOIL Prob.	COILS Position		COILS Prob.	CCHMM-PROF Position		CCHMM-PROF Prob.	CC Pfam prediction	CC SMART prediction
Q8WS23 rols6	1503-1536	1536-1578	upstream TPR-2	81.18%	1552-1580	TPR-2	73.66%	1474-1582	upstream TPR-1 + TPR-2	5.6	1591-1618*	1591-1618*
	1551-1584		TPR-2					1591-1615	TPR-3	5.6		
	1585-1618							1617-1635	downstream TPR-3	6.2		
Q8WS24 rols7	1733-1766	1766-1809	upstream TPR-2	81.18%	1782-1810	TPR-2	73.68%	1704-1812	upstream TPR-1 + TPR-2	5.6	1821-1848*	1821-1848*
	1781-1814		TPR-2					1821-1845	TPR-3	5.6		
	1815-1848							1847-1865	downstream TPR-3	6.2		
Q9C0D5 TANC1	1289-1322	1404-1428	downstream TPR-3	81.80%	1403-1430	downstream TPR-3	96.73%	1257-1400	upstream TPRs + all TPRs	5.9	1403-1430*	1402-1430*
	1336-1369							1402-1427	downstream TPR-3	5.9		
	1370-1403							1800-1831	C-terminus	5.6		
Q9HCD6 TANC2	1244-1277	1352-1377	downstream	12.68%	1352-1378	downstream	50.23%	1214-1293	upstream TPR-1 + TPR-1	5.3	–	–
	1291-1324		TPR-3			TPR-3		1310-1352	upstream TPR-3 +TPR-3	5.9		
	1325-1358							1358-1378	downstream TPR-3	6.2		

Supplementary Table S2: Assessment of coiled-coil regions (CC) compared to TPR positions in TANC proteins and Drosophila homologous (rols). TPR modules are indicated as TPR-1, TPR-2 and TPR-3. Coiled coil predictions were performed with COILS, MARCOIL and CCHMM-PROF. MARCOIL and COILS provide coiled-coil-forming probability obtained in scanning windows of 14, 21 and 28 residues. CCHMM-PROF results display posterior probability of each predicted label (H=coiled coil,.= not coiled-coil) for each position. ***** = predicted E-value is N/A, namely the score is less significant than required threshold.

```
>tpr_c_term_TANC2
ATWAMATSKPDIMIILLSKLMEEGDMFYKKGKVKEAAQRYQYALKKFPREGFGEDLKTFREL
KVSLLLNLSRCRRKMNDFGMAEEFATKALELKPKSYEAYYARARAKRSSRQFAAALEDLNEA
IKLCPNNREIQRLLLRVEEECRQMQQPQQPPPPPQPQQQLPEEAEPEPQHEDIYSVQDIFEE
EYLEQDVENVSIGLQTEARPSQGLPVIQSPPSSPPHRDSAYISSSPLGSHQVFDFRSSSSVG
SPTRQTYQSTSPALSPTHQNSHYRPSPPHTSPAHQGGSYRFSPPPVGGQGKEYPSPPPSPLR
RGPQYRASPPAESMSVYRSQSGSPVRYQQETSVSQLPGRPKSPLSKMAQRPYQMPQLPVAVP
QQGLRLQPAKAQIVRSNQPSPAVHSSTVIPTGAYGQVAHSMASKYQSSQGDIGVSQSRLVYQ
GSIGGIVGDGRPVQHVQASLSAGAICQHGGLTKEDLPQRPSSAYRGGVRYSQTPQIGRSQSA
SYYPVCHSKLDLERSSSQLGSPDVSHLIRRPISVNPNEIKPHPPTPRPLLHSQSVGLRFSPS
SNSISSTSNLTPTFRPSSSIQQMEIPLKPAYERSCDELSPVSPTQGGYPSEPTRSRTTPFMG
IIDKTARTQQYPHLHQQNRTWAVSSVDTVLSPTSPGNLPQPESFSPPSSISNIAFYNKTNNA
QNGHLLEDDYYSPHGMLANGSRGDLLERVSQASSYPDVKVARTLPVAQAYQDNLYRQLSRDS
RQGQTSPIKPKRPFVESNV

The sequence name : tpr+c-term_TANC2
Per-protein P-value for being TPR : 9.3E-21
Probability for being TPR : 100.00%

Repeat Begin               Alignment                  End    P-value
-------------------------------------------------------------------
TPR    17       LSKLMEEGDMFYKKGKVKEAAQRYQYALKKFPRE   50     1.7e-04
TPR    64       VSLLLNLSRCRRKMNDFGMAEEFATKALELKPKS   97     7.7e-11
TPR    98       YEAYYARARAKRSSRQFAAALEDLNEAIKLCPNN   131    1.5e-10



>tpr_c_term_TANC1
AAWAMATSKPDILIILLQKLMEEGNVMYKKGKMKEAAQRYQYALRKFPREGFGEDMRPFNEL
RVSLYLNLSRCRRKTNDFGMAEEFASKALELKPKSYEAFYARARAKRNSRQFVAALADLQEA
VKLCPTNQEVKRLLARVEEECKQLQRSQQQKQQGPLPAPLNDSENEEDTPTPGLSDHFHSEE
TEEEETSPQEESVSPTPRSQPSSSVPSSYIRNLQEGLQSKGRPVSPQSRAGIGKSLREPVAQ
PGLLLQPSKQAQIVKTSQHLGSGQSAVRNGSMKVQISSQNPPPSPMPGRIAATPAGSRTQHL
EGTGTFTTRAGCGHFGDRLGPSQNVRLQCGENGPAHPLPSKTKTTERLLSHSSVAVDAAPPN
QGGLATCSDVRHPASLTSSGSSGSPSSSIKMSSSTSSLTSSSSFSDGFKVQGPDTRIKDKVV
THVQSGTAEHRPRNTPFMGIMDKTARFQQQSNPPSRSWHCPAPEGLLTNTSSAAGLQSANTE
KPSLMQVGGYNNQAKTCSVSTLSASVHNGAQVKELEESKCQIPVHSQENRITKTVSHLYQES
ISKQQPHISNEAHRSHLTAAKPKRSFIESNV


The sequence name : tpr_c_term_TANC1
Per-protein P-value for being TPR : 3.2E-20
Probability for being TPR : 100.00%

Repeat Begin               Alignment                  End    P-value
-------------------------------------------------------------------
TPR    17       LQKLMEEGNVMYKKGKMKEAAQRYQYALRKFPRE   50     6.5e-05
TPR    64       VSLYLNLSRCRRKTNDFGMAEEFASKALELKPKS   97     2.6e-10
TPR    98       YEAFYARARAKRNSRQFVAALADLQEAVKLCPTN   131    3.7e-09
TPR    132      QEVKRLLARVEEECKQLQRSQQQKQQGPLPAPLN   165    3.6e-02
TPR    172      DTPTPGLSDHFHSEETEEEETSPQEESVSPTPRS   205    1.5e-01
TPR    213      SSYIRNLQEGLQSKGRPVSPQSRAGIGKSLREPV   246    2.4e-01
TPR    255      PSKQAQIVKTSQHLGSGQSAVRNGSMKVQISSQN   288    8.2e-03
```

Supplementary table S3. TPR-pred results for TANC1 and TANC2

Variant	AA change	SIFT	Provean	Polyphen2		SNAP2	Mupro	I-Mutant2.0	Align-GVGD	Mutation Taster	Mutation Assesor	PhD-SNP	UMD-Predictor	D/tot
		Prediction	Prediction. Score	HumDiv (effect score)	HumVar (effect score)	Variant. Effect. Score Accuracy	Effect (Stability) Confidence score	Prediction. Reliability index	Prediction Score	Prediction, Score	Func. Impact	Prediction, Accuracy	Prediction, Prob.	
ch17.61432669 C/T[a]	R760C	not tolerated	Deleterious -5.593	probably damaging 1.000	probably damaging 0.999	R760C effect 54.75%	DECREASE -0.897	Increase Stability. 2	most likely. 179.53	disease_causing. 0.999999992696	Medium	Deleterious. 73%	Pathogenic. 99%	11/12
ch17.61457099 C/T[b]	A794V	not tolerated	Deleterious -3.669	probably damaging . 1.000	probably damaging. 0.980	A794V effect 29.63%	DECREASE -0.645	Decrease Stability. 7	most likely. 65.28	disease_causing. 0.999999963786	Medium	Deleterious. 77%	Pathogenic. 75%	12/12
ch17.61498409 A/G[c]	H1689 R	tolerated	Deleterious -3.107	possibly damaging 0.932	possibly damaging 0.840	H1689R neutral -12.57%	INCREASE 0.746	Decrease Stability. 3	less likely. 0.00	disease_causing. 0.999908818377	Low	Neutral. 78%	Pathogenic. 78%	6/12

Supplementary table S4: Pathogenicity prediction of known missense mutations in TANC2 sequence

TANC2 mutations described in literature: **a.** de Ligt et al. 2012; **b.** Fromer et al. 2014; **c.** Iossifov et al. 2012. Twelve sequence-based prediction tool were employed to assess the effects of the mutations on TANC2 function. When available, the related score and/or the accuracy for each prediction are reported. **D/tot:** the ratio between "damaging" prediction and their total number.

Supplementary Figures

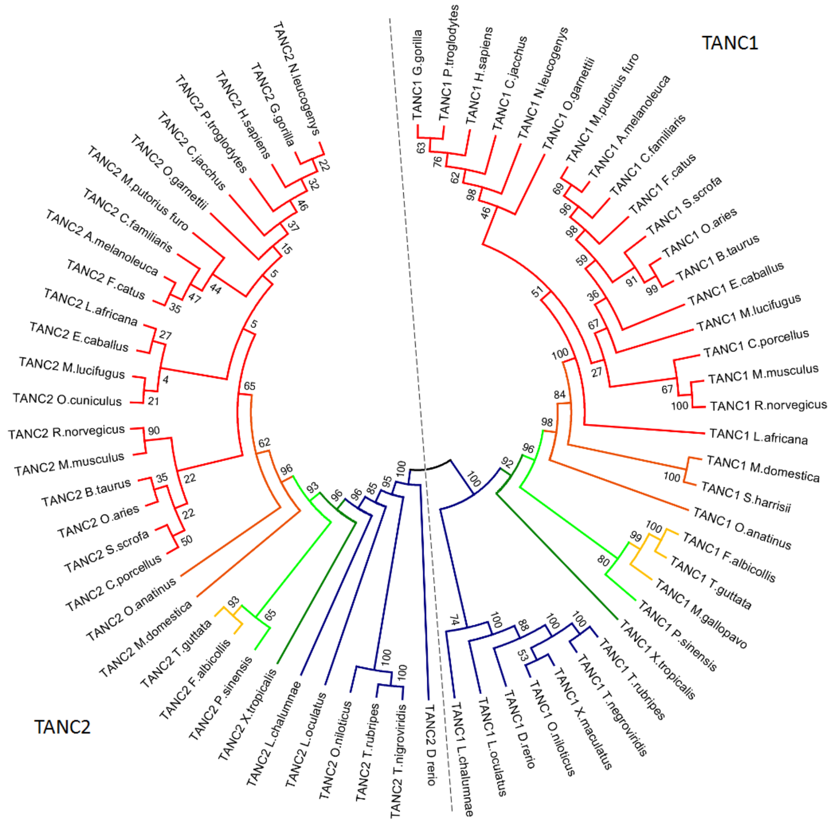

Supplementary Figure S1: Phylogenetic tree reconstruction.

The tree is divided in three main clusters: TANC2 orthologs, TANC1 orthologs and species without TANC gene duplication. The latter are Arthropoda (*Strigamia maritima* and *Ixodes scapularis*) in blue, insects in purple, *Ciona intestinalis* (Tunicata) in grey and *Trichinella spiralis* (Nematoda) in black. For each cluster, six main groups can be recognized: fish (blue), amphibious (dark green, *Xenopus tropicalis*), reptile (green, *Pelodiscus sinensis*), bird (yellow), the marsupial *Monodephis domestica* and *Ornithorhynchus anatinus* (orange), and mammals (red).

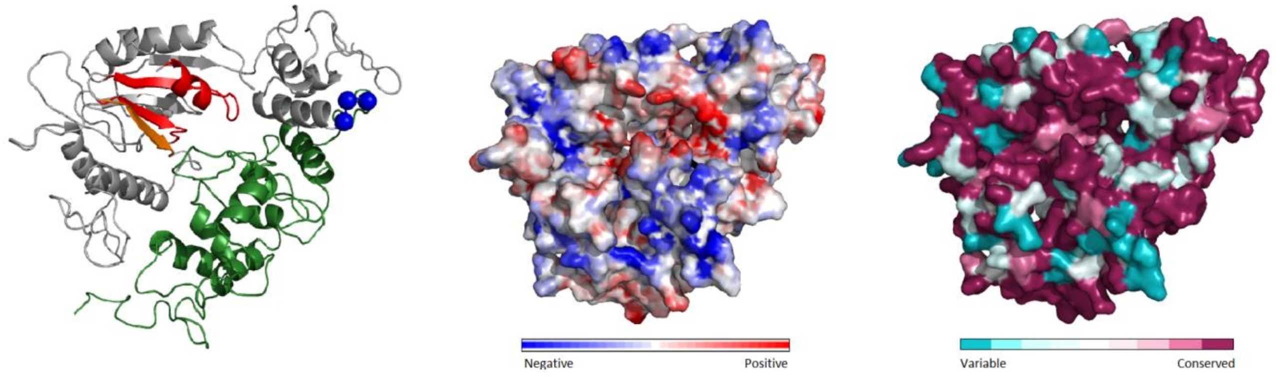

Supplementary Figure 2: Structural analysis of ATPase domain in TANC2.

Cartoon of TANC2 ATPase domain model (front part) is coloured as following: Walker motifs is in red, ASCE in orange, HETHS domain in green, GxP motif in blue spheres. Electrostatic properties of front surfaces are shown: negative charges in blue and red charges in red. Consurf analysis of front surfaces, colour code from unconserved (cyan) to conserved (purple) residues.

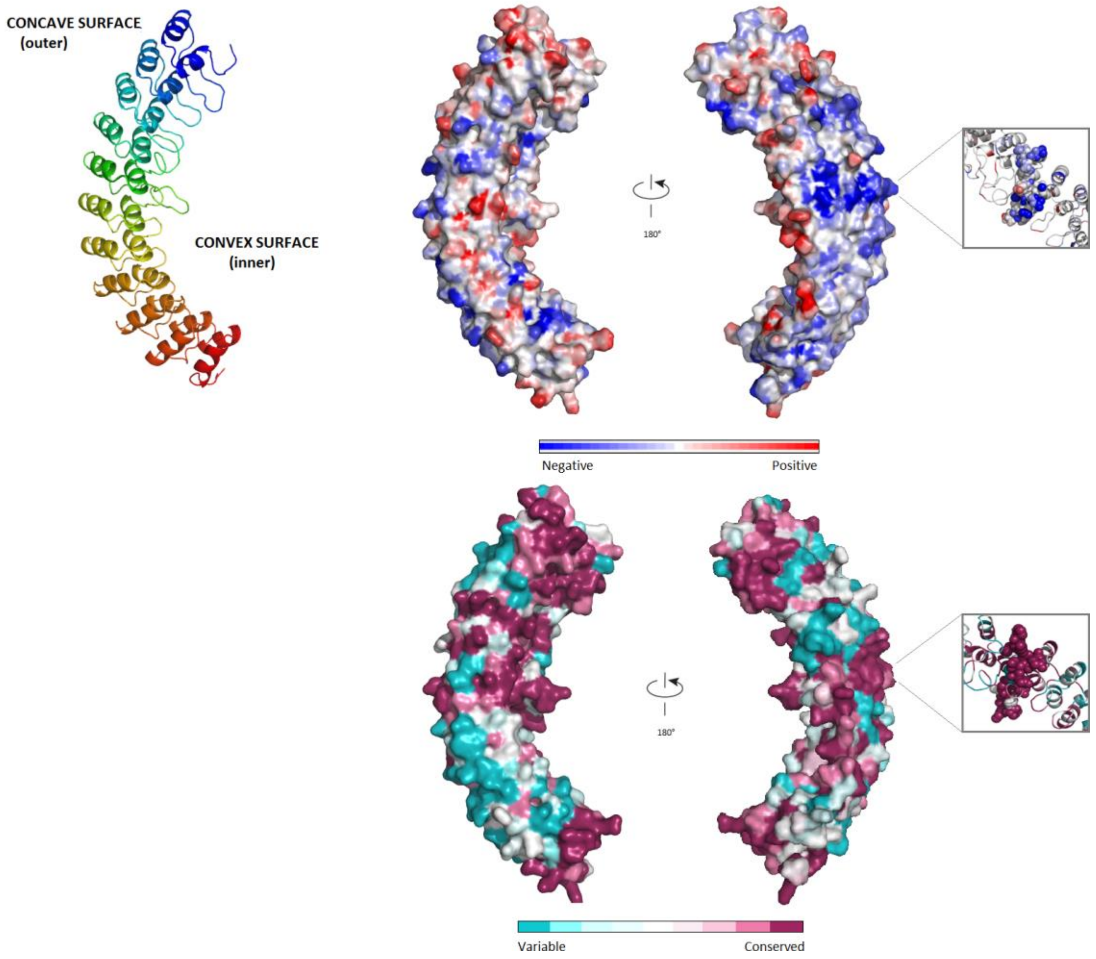

Supplementary Figure 3: Structural analysis of ankyrin domain in TANC2.

Cartoon of TANC2 AR domain model is coloured from N-terminus (blue) to C-terminus (red). Electrostatic properties of turn-loop surfaces and connecting-loop surfaces are shown: negative charges in blue and red charges in red. Consurf analysis of turn-loop surface and connecting-loop surface, colour code from unconserved (cyan) to conserved (purple) residues. The magnification of connecting loop between the fifth and the sixth repeats is shown in the box.

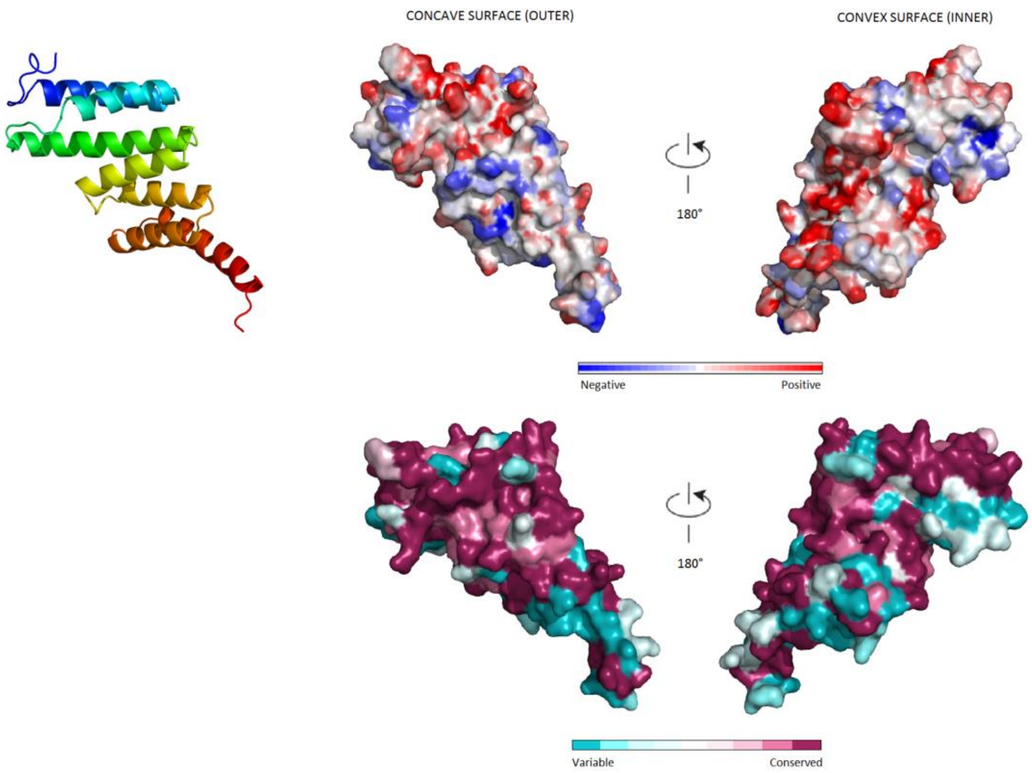

Supplementary Figure 4: Structural analysis of TPR domain in TANC2.

Cartoon of TANC2 TPR domain model are coloured from N-terminus (blue) to C-terminus (red). Electrostatic properties of concave and convex surfaces are shown: negative charges in blue and red charges in red. Consurf analysis of turn-loop surfaces and connecting-loop surfaces, colour code from unconserved (cyan) to conserved (purple) residues.

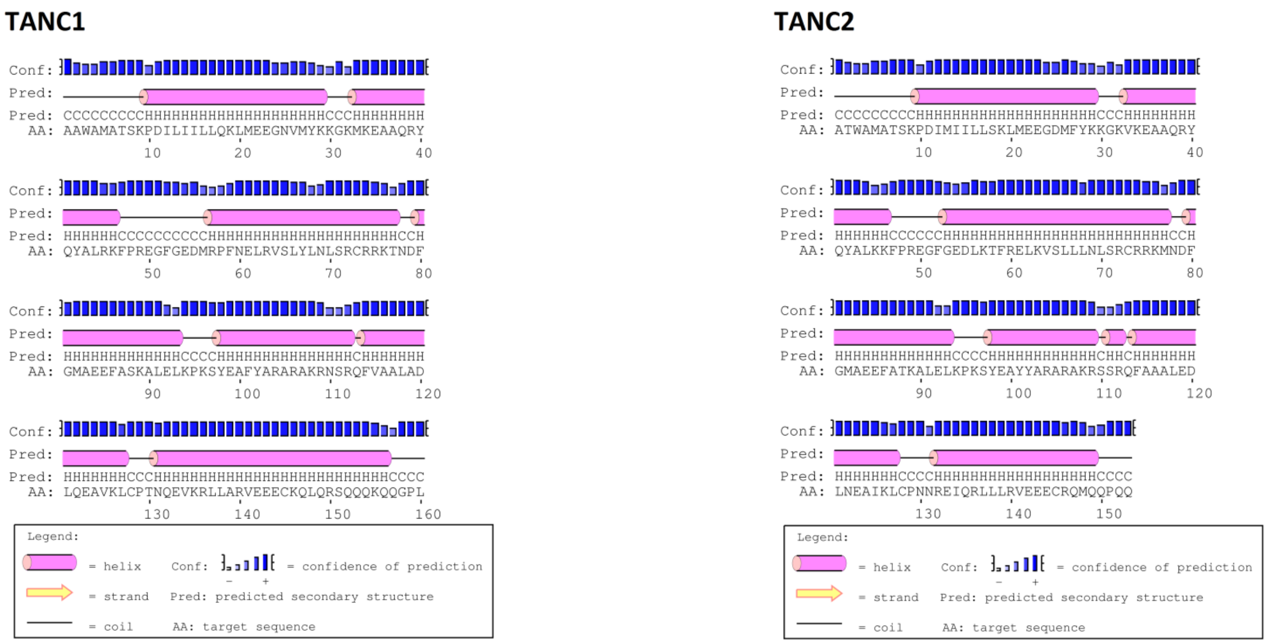

Supplementary Figure 5: Schematic representation of TANC secondary structure at TPR regions (PSIPRED).

A **B**

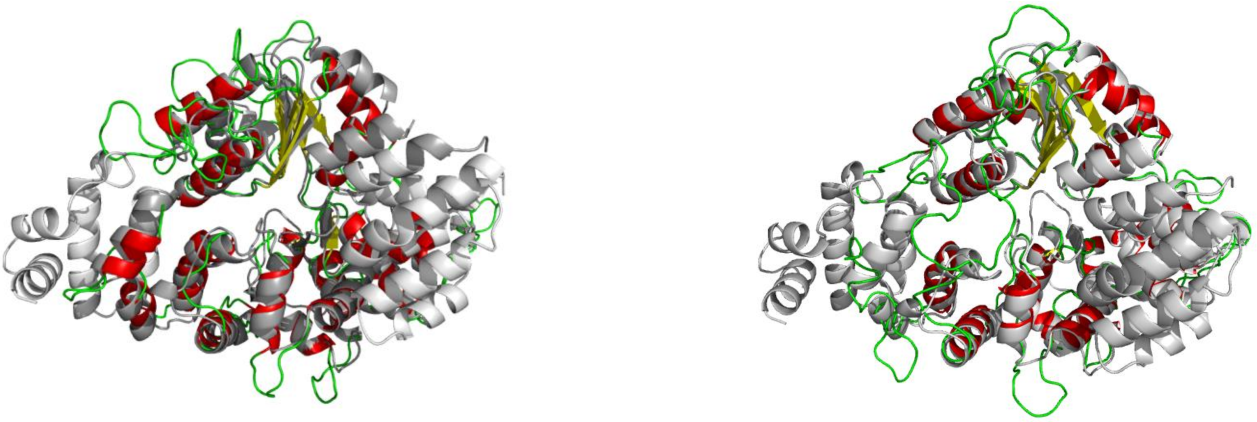

Supplementary Figure 6: Superposition of TANC ATPase region, coloured according secondary structure (red = alpha-helices, green = loops and yellow = beta-strands) , and template (1zt6 = apoptotic protease-activating factor 1, APAF1), in grey. **A**: TANC1 and 1z6t superposition; **B**: TANC2 and 1z6t superposition. In both cases, the TANC ATPAse signature motifs are superimposed to template catalytic core.
